# Perceived Stigma and Fear of Unintended Disclosure are Barriers in Medication Adherence in Adolescents with Perinatal HIV in Botswana: A Qualitative Study

**DOI:** 10.1155/2019/9623159

**Published:** 2019-12-02

**Authors:** Sphiwe Madiba, Unaswi Josiah

**Affiliations:** Department of Public Health, Sefako Makgatho Health Sciences University, Pretoria, South Africa

## Abstract

**Background:**

Maintaining optimal adherence to antiretroviral treatment (ART) is a challenge for adolescents with perinatally HIV (ALPHIV), and there is little consensus on what factors contribute to adherence in this population. This study assessed self-reported medication adherence among ALPHIV and explored structural factors that hinder or motivate them to adhere.

**Methods:**

This qualitative study used in-depth interviews with ALPHIV at the infectious disease control centre of a teaching hospital in Botswana. Thirty adolescents aged 12–19 years who were aware of their HIV status were recruited purposively. Transcribed interviews were analysed using the thematic approach and NVivo data analysis software.

**Findings:**

Nonadherence was a problem across age groups and gender. Perceived stigma was a major barrier to ART adherence. The fear of stigma and unintended disclosure were more pronounced in those attending boarding school. The adolescents were not willing to take medication in front of roommates and outside of the home. They opted for hiding and taking medication in privacy which led to missed doses. The heightened fear of being seen collecting ART medication affected keeping appointments for clinic visits. Fear of stigma also influenced the choice of action when there was a clash between school activities, dosing times, and scheduled clinic appointments for ART refill. The home environment was the main facilitator for adherence. Support was the strongest motivator for adolescents to adhere and keep up with clinic visits. On a personal level, the desire to be healthy and live long was a major motivator to adhere.

**Conclusions:**

The fear of stigma shaped the adolescents' adherence behaviour. Perceived stigma affected the time and place to take medication, the visit to the clinic for ART refill, and self-disclosure of HIV status. There is need to encourage adolescents to self-disclose their HIV status to friends since the fear of unintended disclosure fuelled perceived stigma. Planning of clinic appointments should also be consistent with realistic daily activities of adolescents.

## 1. Introduction

The successful treatment of children with perinatal HIV has increased the number of adolescents on antiretroviral treatment (ART) globally. The World Health Organization (WHO) defines an adolescent as any person between ages 10 and 19 [1]. Of the global population of adolescents living with perinatally acquired HIV (ALPHIV), 79% live in sub-Saharan Africa (SSA) [2]. However, sustaining optimal ART adherence for ALPHIV has emerged as a major challenge for survival, health, and prevention of sexual transmission of HIV [3, 4]. There is substantial evidence that, when compared with younger children and adults, ALPHIV experience greater challenges in terms of treatment adherence and have disproportionately higher rates of virological failure [5–10]. A recent systematic review of studies conducted with ALPHIV in low income countries reported adherence rates of 16 to 99% [3].

Nonadherence to ART causes worse outcomes in ALPHIV than HIV-infected adults [11, 12]. In ALPHIV, nonadherence is associated with treatment failure, emergence of drug resistance, clinical progression of disease, high hospitalization rate, high morbidity, and increases the risk of onward horizontal transmission of drug-resistant strains when adolescents become sexually active [13, 14]. Almost a quarter of ALPHIV fail the second-line therapy within 12 months because of poor adherence rather than drug resistance [15]. Many countries in SSA have limited ART drug options and formulations; therefore, maintaining high rates of adherence to the first-line medication regimen is crucial for successful treatment of HIV to ensure optimal treatment benefits for adolescents [10, 16].

Virological suppression requires effective ART, which implies the correct dose of appropriate ART being consistently and correctly taken [10]. In the case of ALPHIV, adhering requires an understanding about why they have to take ART, how it works, and how it benefits them [17]. This suggests that poor adherence could be because ALPHIV lack the information they need to understand HIV and its treatment or the reasons they were taking medication [18–20]. In addition, Garvie et al. [21] point out that adherence is not merely remembering taking prescribed medication but also following strict dosage instructions, timing intervals, and dietary guidelines to which ALPHIV must also adhere. Therefore, ALPHIV have worse adherence not only because of their unique behavioural characteristics and lifestyles but also because of the complex nature of ART medication and expectations for adherence. The desire for conformity with peers during adolescence and fear of being treated differently and being stigmatized because of their HIV status further complicate the situation and affect adherence [22, 23].

Factors associated with poor ART adherence among ALPHIV are multifactorial and include family situations, socioeconomic factors, drug-related issues, and community issues [19, 24]. The most cited drug-related barriers to good adherence include drug fatigue, inconvenient dosing frequency, complex drug regimens, undesirable side effects, and clinic appointment schedules that interfere with schooling [6, 18]. Socioeconomic factors such as poor housing, financial burden, lack of food, and lack of transport money to access the clinic affect adherence negatively [25]. At the personal level, fear of disclosure and stigma by peers is the most prominent barrier to adherence among ALPHIV [24, 26, 27].

The poor adherence to ART among ALPHIV underscores the need to identify risk factors that contribute to nonadherence in this population in SSA, including Botswana [9]. However, very few studies have targeted ART adherence among adolescents [6, 16, 26], which is often more complex and unique to them compared to adults [23]. Furthermore, there is little consensus on what factors contribute most to ART adherence in children and adolescents in SSA [28]. Therefore, this study was conducted to assess self-reported medication adherence among ALPHIV and explore structural factors that hinder or motivate them to adhere to ART. More information is needed on ART and the challenges faced by adolescents in order to develop appropriate interventions to improve their adherence levels and health outcomes [29]. Optimal adherence is essential to ensure treatment success and prevent onward HIV transmission when the adolescents become sexually active [10].

## 2. Methods

### 2.1. Study Design and Population

This study used a qualitative research design using in-depth interviews with ALPHIV at the infectious disease control centre (IDDC) in a multispecialty tertiary care and teaching hospital in Francistown, Botswana. The study was conducted between October 2015 and January 2016.

The IDCC is a specialized centre offering HIV services such viral load and CD4 count monitoring, managing opportunistic infections and HIV-related complications, and ART adherence counselling. It is one of two referral facilities for first initiation of ART and monitoring of those who need specialized treatment such as very sick patients, relapses, and treatment failure. At the time of the study, approximately 1500 ALPHIV were enrolled for ART services in the centre and records show that about 40% of ALPHIV were not adhering, despite the fact that protocols for ART adherence are in place. Eligible adolescents were recruited when they came to the IDCC for follow-up, came to routine visits for ART refill, or came to attend the failure clinic that the IDCC runs for nonadherent patients. To recruit adolescents who were minors, the second author and a trained research assistant (researchers) approached the caregivers individually to establish whether the child was aware of their HIV status and to ask for permission to approach their children to participate in the study. They explained the objectives of the study, the interview procedures, informed consent, assent, confidentiality, and voluntary participation. Adolescents whose caregivers gave permission were approached in the presence of the caregiver and asked for assent to participate in the study [30]. The adolescents were informed that they would be privately interviewed in the absence of their caregivers.

Purposeful sampling was used to ensure a broad distribution of adolescents on ART. In purposeful sampling, the researcher selects participants who can offer a meaningful perspective on the phenomenon of interest [31]. The researcher further employed maximum variation sampling to ensure that the sample reflects a diverse group of adolescents to make sure there is variation in the information [32]. For example, the study ensured that the views of both boys and girls, younger and older, as well as adherent and nonadherent adolescents were captured. The adolescents were sampled if they were perinatally infected, were aged 12–19 years old, had been taking ART at the study site for more than 12 months, and were aware of their HIV status. The clinic staff who knew the adolescents well were instrumental in identifying adolescents who met the eligibility criteria and informed the purposeful selection of adolescents into adherence and nonadherence groups. As mentioned, IDCC runs a failure clinic for patients who are not adhering to their ART, the nonadherent adolescents were recruited from this clinic, and it was confirmed that they were not adhering to their medication. Furthermore, awareness of HIV status was confirmed with the caregivers and the clinic staff who keep records of children who have been informed about their HIV serostatus. As a principle, all the adolescents who attend the failure clinic are informed about their HIV serostatus during counselling. As the aim of the study was to explore structural factors that hinder or motivate adherence to ART, the allocation of adolescents to adherence and nonadherence groups was based on clinic and patients' records. A total of 30 adolescents were interviewed. The sample size was guided by the richness of data and data saturation [33]. Furthermore, previous studies on ART adherence had reached a saturation of themes after interviewing from 20 to 50 children and adolescents [34].

### 2.2. Data Collection

In-depth interviews (IDIs) were conducted by the second author (UJ) and a research assistant trained in the objectives of the study by the lead author. An English interview guide with open-ended questions was translated into Setswana and used to explore adolescents' self-reported adherence as well as the facilitators and barriers to adhere to ART. The use of IDs coupled with the use of Setswana and English, depending on the wishes of the adolescents, allowed a rich understanding of what hinders adolescents or motivates them to adhere. In addition, the researchers made use of probes and follow-up questions in order to engage intensely with the participants. The use of the local language allowed them to speak freely about their experiences. The participants were interviewed in a consultation room to ensure privacy. Each interview was conducted in the absence of the caregiver and a digital recorder was used with consent. Prior to each interview, the researchers obtained informed written consent and assent and explained the use of a digital recorder. Each interview lasted approximately 20–35 minutes and they were done after the adolescents had finished their medical check-ups to avoid disruption of the clinic routine.

At the end of the interview, sociodemographic variables including age, gender, school grade, the duration that the adolescents had known their HIV status, and duration on ART were captured.

### 2.3. Data Analysis

Thematic analysis was done following the approach of Braun and Clarke [35] using both a deductive and an inductive approach to identify and report patterns or themes within the data. The audio recordings were transcribed verbatim and translated into English by the second author (UJ) and the research assistant who conducted some of the interviews. To ensure data quality, UJ played the recordings to correct inaccuracies and inconsistencies in order to verify the transcripts. Both authors performed the analyses and interpretation of data to enhance credibility of the study findings. The authors independently read transcripts repeatedly to familiarise themselves with the data and identify emergent codes. The authors then discussed and agreed on initial codes that reflect adherence facilitators and barriers. This culminated in the development of a codebook to aid the analysis of data. NVivo 10, which is qualitative data analysis software, was used to code the transcripts. The codebook was updated as the authors identified new themes. The final themes were decided upon by agreement between the authors and were used to present the data.

### 2.4. Rigour

A number of strategies and methodologies were used to enhance rigour and establish credibility of the study findings. We used the methodological triangulation as one of the strategies to demonstrate credibility. In the within-method triangulation, researchers use multiple methods of data collections to collect data on the same topic [36–38]. We conducted interviews, extracted clinical data from records, captured demographic and HIV-related quantitative data, and documented detailed field notes of all the observations during the interviews. Methodological triangulation was particularly important to confirm self-reported adherence or nonadherence with the clinical information extracted from the clinical records to increase credibility. In addition, we employed investigator triangulation as an important concept in data analysis to mitigate investigators bias [39]. All the authors were involved in the coding, analyses, and interpretation of the data.

### 2.5. Ethical Considerations

The study was approved by the Research Ethics Committee of Sefako Makgatho Health Sciences (SMUREC/104/2017: PG) and the Research Ethics Committee of the Republic of Botswana. The management of the hospital also granted permission to conduct the study. Adolescents 18 years and above signed their own informed consent and caregivers signed the consent for adolescents aged 12–17 years. Those below the age of 18 years also provided written assent in addition to their caregivers' consent. Assent from the adolescents was obtained in accordance with the National and International Guiding Principles for conducting research with minors, and the procedure for consenting was conducted in the local language [30, 40]. Furthermore, the study addresses privacy and confidentiality consistent with guidelines for research involving minors [30]. The researchers confirmed with the caregivers and clinic staff that all the adolescents that were approached to participate in the study were aware of their HIV status. Pseudonyms were used during the interviews to protect the identity of the adolescents. The researchers explained voluntary participation and withdrawal from the study to the adolescents and the caregivers to minimize the pressure to participate.

## 3. Findings

### 3.1. Description of Study Sample

**Figure 1:**
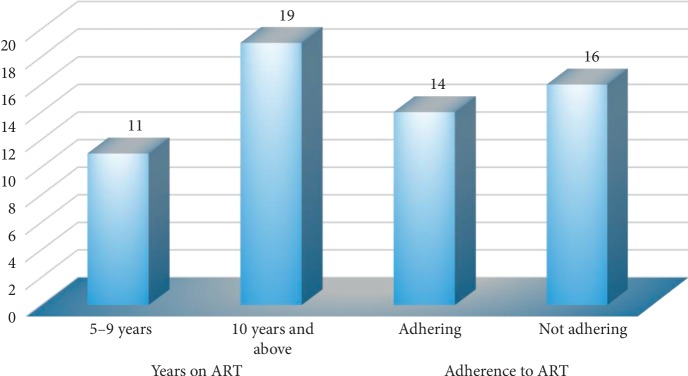
Antiretroviral treatment and clinical data of adolescents with perinatal HIV.

Thirty adolescents with PHIV were interviewed, and Table 1 presents their demographics data. Their ages ranged from 12 to 19 years with a mean age of 15.7 years. There were 17 males compared to 13 females and 18 were in secondary school. Eleven adolescents were orphans under the care of grandmothers, siblings such as older sisters, aunts, and social workers. All the adolescents were aware of their HIV status, and 19 have been on ART for 10 years and 16 reported that they were not adhering and were attending the failure clinic and some had started second-line treatment (Figure 1).

### 3.2. Themes

Table 2 shows a summary of the two main themes—barriers and facilitators of adherence—and eight subthemes that emerged from the analysis of the in-depth interviews. The subthemes include (1) lack of transport money, (2) fear of stigma and discrimination, (3) clash between school and treatment schedule, (4) treatment side effects, (5) living arrangements, (6) understanding the benefits of ART, (7) fear of getting sick and dying, and (8) making ART a part of daily life.

#### 3.2.1. Barriers to Adhere to ART

Barriers to adhere to ART were multifactorial and included the household, individual, school, and health system levels. Lack of transport money, fear of stigma and discrimination, living in a boarding school, clash between school and treatment schedules, and treatment side effects emerged as major barriers to adhere to ART medication among ALPHIV.

*(1) Lack of Transport Money.* The cost of transport emerged as one of the major barriers for adherence among the study sample. The adolescents often missed their monthly check-ups and ART refill because they did not have transport money to get to the IDCC before their medication ran out. Most of them lived far from the IDCC and the lack of transport money resulted in travelling long distances to and from the centre.

I walk to the health facility because of lack of money for transport (Vusa, 15-year-old male).

To come here every month is a big challenge because of lack of transport money (Boyce, 18-year-old male).

I fail to keep appointments because there will be no transport money and we end up borrowing (Lebo, 19-year-old female).

*(2) Fear of Stigma and Discrimination.* Perceived stigma and discrimination deterred the adolescents from obtaining, taking, and keeping their medications. They described how they would avoid going to the clinic to obtain their ART because they did not want to be seen by their peers at the clinic collecting ART medication and in so doing link them to HIV diagnosis. They further described how they avoided taking pills in front of others for fear of stigma, rejection, and gossip by their peers.

I don't prefer boarding school because I am afraid to be seen and to be laughed at, that is why I take my medication in private. Containers make noise and people will know at boarding school, because no one knows (her HIV status) (Hope, 15-year-old female).

I don't want my friends to know, I am scared to take them (ART) in front of other kids and I cannot take them anywhere. I want to take them but at home only (Lorato 17-year-old female).

I don't want to take my medication in front of people may be they will ask questions (Polka, 13-year-old male).

*(3) Living in a Boarding School.*Eleven adolescents were orphans and were taken care of by social workers. Ten of the orphans were living in boarding schools. Adherence was a challenge for those who live in boarding schools. Many adolescents were challenged by a lack of privacy because they had to share accommodation with others in hostels. They lacked a private place to keep their medication so that they would not be found out or seen by their roommates. They also often lacked a private place to take their medication which led to missed doses and this negatively affected their adherence.

I have to hide my medication at school for secrecy (Rex, 17-year-old male).

During sports I pack my medication in a paracetamol labelled sachet (Thero, 16-year-old male).

When I am at school I prepare my medication before everyone and put them in a pocket and when I brush my teeth I take them (Hope, 15-year-old female).

*(4) Clash between School and Treatment Schedule.* The school setting presented a unique barrier to adherence to medication for the adolescents. Most (27 out of 30) were attending secondary and high school and indicated that it was not easy to leave school to keep clinic appointments for ART refill, particularly because they did not tell their teachers about their HIV status. They further experienced difficulties to keep to their school timetable and honour clinic appointments when there were conflicts between their medication schedules and their class schedules.

The set dates for my clinic means that I have to miss writing tests and examination and I do not like writing alone (Hope, 15-year-old female).

I miss appointments when I am writing tests or examination. I end up coming to change the scheduled dates (Keba, 14-year-old female).

About appointments most of the time I tell them before examination or school projects, then ask for time before or after so that I can write (Thero, 16-year-old male).

Furthermore, the adolescents identified issues around remembering to take ART when their daily home and school activities change. They found it difficult to adhere to medication when they attempted to fit the time to take medication to their school schedules like playing sports, school trips, and school and/or early morning study schedules.

I am released late from school and reach home past the time I am supposed to take my medication. In the morning, I leave for school as early as 5am, before 6am the time I am supposed to take my medication and end up taking them late (Fraser, 15-year-old male).

At school we have boot camp, and when I go there, I come home late and during examination times, I wake up early and fail to take my medication as prescribed (Pearl, 18-year-old female).

*(5) Treatment Side Effects.* Treatment side effects were a major barrier to adherence among adolescents. Some of the adolescents were attending the ART failure clinic and had started the second-line ART regimen. The adolescents experienced unbearable ART side effects and felt that the pills were too many and too big.

The pill causes dizziness, nausea, no taste and I sleep most of the time in class (Gladys, 15-year-old female).

Alluvia is too big, if you take it, you have to eat a sweet so that the smell doesn't affect you because you will end up vomiting, and the colour of Alluvia and smell affect me. They make me feel dizzy when I take them on an empty stomach (Vusa, 15-year-old male).

The pills are too many…, if I feel they are too many I take a few and leave others (Johnny, 13-year-old male).

I take six pills in the morning and four in the evening. They are many and have side effects. I really don't like Alluvia because it makes me nauseas (John, 17-year-old male).

The treatment side effects were exaggerated when there was no food needed to take with the pills. The adolescents experienced discomfort, dizziness, and vomiting when they took their medication without food. This led them not to take their medication when food was not available.

There is shortage of food at home and when there is no food I don't take them (Kabo, 19-year-old male).

Sometimes there will be no food and then I have to take my medication on an empty stomach (Pako, 18-year-old male).

#### 3.2.2. Facilitators of ART Adherence

Living arrangements, understanding the benefits of ART, fear of getting sick and dying, and making ART a part of daily life were subthemes that explain what motivates children and adolescents with PHIV to adhere to medication.

*(1) Living Arrangements*. Living with their own family provides a supportive environment for adolescents to adhere. In their narratives, they indicated that their families, particularly parents and siblings, supported them. The most important way the adolescents received support from their family members was in reminders to take pills, accompaniment to the clinic, emotional support, and counselling on the reasons to take the medication.

My mother helps me; sometimes when I am sleeping she wakes me up and give me my pills. The other thing is that my sister also helps me (Keba, 14-year-old female).

I show my aunt at home that I am now taking the pills and she will be watching me swallow them (Nkele, 16-year-old female).

My mother supports me... I always come to the clinic with my mother (Alicia, 14-year-old female).

*(2) Understanding the Benefits of ART*. Improved levels of adherence were related to positive beliefs about ART. The adolescents' belief that ARVs protect them from acquiring opportunistic infections motivated them to adhere. Most of the adolescents have seen significant improvement in their health after initiating ART which led to increased trust in the ART medications.

I understand that these pills protect my life because if I do not take them it's the end of my life (Kate, 16-year-old female).

If I don't follow instructions the virus will multiply and I will develop drug intolerance. The pill won't be effective in fighting the virus if I miss doses. I will get sick while taking my medication (Mike, 15-year-old male).

I was taught how to take medication and why I should take them. When I skip my medication there will be consequences of low CD4 count (Uyapo, 13-year-old male).

The treatment is my life (Dumisani, 18-year-old male).

*(3) Fear of Getting Sick and Dying*. Understanding the need to adhere and the fear of getting sick from poor adherence to ART was a major motivator for adherence among adolescents. For most of the adolescents, the benefit of taking their medication correctly was seen as a way to live as normal life as their HIV uninfected peers.

I don't want to end up being sick (Boyce, 18-year-old male).

I don't want to contact diseases such as TB and other viruses (Lindi, 19-year-old female).

If I don't take my pills correctly I will end up getting sick. These pills are my life. If I don't take them I will die, because they suppress the virus and that I should not contact diseases such as TB and the like (Jean, 18-year-old male).

I was told that it's a must to take my pills so that I may live long, taking them at the right time (Mike, 15-year-old male).

*(4) Making ART a Part of Daily Life*. The adolescents with the support from their families found ways to incorporate taking medication into their lifestyles. Those who had disclosed their HIV status to teachers and friends and could link ART to their daily school routines found it much easier to remember to take it correctly.

I don't have challenges…, my mother informed my teacher and she puts my medication separately and teaches me how to take them (Thandeka, 13-year-old female).

My mother tells me that even if I go to school trips, my pills are the first priority, well…, I attend counselling and they teach me different things. My table tennis coach also reminds me to take my medication (Mike, 15-year-old male).

About sports and school trips my guidance teacher and my friends who are on the same treatments reminds me (Gladys, 15-year-old female).

## 4. Discussion

This study has shown that there are various problems that affect adolescents' adherence to ART medication. Sixteen adolescents were not adhering and some have started the second-line ART regimen. The problem of nonadherence was common among all the age groups with both younger and older adolescents being nonadherent. The study found that adolescents experienced adherence problems at the household, school, and health facility levels.

At the household level, the barriers to ART adherence were multifactorial and included living arrangements, family support, and socioeconomic factors [24]. At the household level, living arrangements were identified as both barriers and facilitators of adherence. The home is critical in providing an environment to optimising adherence among ALPHIV. Those who reported less difficulty in adhering to medication discussed how their families reminded them, supervised them to take their medication, and ensured that they had their medication if they went on school trips. The findings are consistent with studies done in other parts of SSA where the family was identified as the source of emotional and instrumental support for adherence [23, 24, 26, 27]. Having a clinic companion also emerged as a positive influence on adherence. The presence of a caregiver actively participating in the adolescent's HIV care and treatment contributed to better adherence to ART [41].

The school was identified as an unfavourable environment and a hindrance to adherence among ALPHIV. The adolescents missed their clinic appointments because of clashes between school activities such as class tests or exams and the scheduled clinic appointments. Clinic appointments that interfered with schooling schedules were identified as barriers to adherence in previous studies [6, 27]. The study found that the adolescents chose the school activities over clinic appointments which resulted in not collecting their ART on time. Even when the adolescents were given an opportunity to write the exam alone at a different time or date, they did not find that acceptable as this would generate unwanted questions from their peers who were unaware of their serostatus. The fear of unintended disclosure and stigma and discrimination influenced their decision to a large extent [27].

Any disruptions in the routine dosing time such as after school activities and sporting events which kept the adolescents from their homes affected adherence. For example, when adolescents are released late from school, they miss the evening dosing time, and if they leave for school too early, they miss the morning doses. This shows that the competing demands between taking ART and managing school schedules often resulted in skipping and missing dosing times [27, 29]. Therefore, it is important that treatment schedules link to realistic daily activities of adolescents [16].

The boarding school was a major structural barrier to adherence among ALPHIV. Those who live in boarding schools lacked a parental figure and family for support. The study found poor adherence among adolescents who reported living in boarding schools. The absence of an adult presented a problem for adolescents who need support with lifelong treatment and to access appointments for check-up and ART refill [23, 29]. Furthermore, living in boarding school was a barrier to adherence since those in boarding schools lacked privacy to take their medication, which led to missing doses [24]. The adolescents were afraid of taking their medication in front of their roommates because of fear of unintentional disclosure of HIV status and subsequent stigma, discrimination, isolation, and rejection. Perceived stigmatization following disclosure was reported as adversely affecting the way adolescents take their medication in other studies [7, 23, 26, 27].

Whereas the adolescents in this study were afraid of perceived stigma and discrimination, a study conducted in Tanzania reported that ALPHIV experienced mocking and segregation within the school environment which made it hard for them to adhere [29]. Perceived stigma resulted in fear of disclosure to roommates who might have offered support and reminded them to take their ART. Instead, the adolescents had to hide their medication from their roommates and they could not take ART in the hostels resulting in missing doses or not taking medication at the right time [7, 26, 29].

Accessing the IDCC was hindered by the lack of money for transportation, which was worse for adolescents who resided in remote areas. As reported in previous studies, distance and lack of transportation money were barriers to adherence and affected the ability of adolescents to honour their clinic appointments for ART refill [18, 42, 43]. Clinic appointments for ART refill were further affected by the fear of being seen collecting ART medication and possibly being labelled as HIV positive [27, 29]. The desire to conform during adolescence and fear of being treated differently and being stigmatized because of their HIV status affect treatment adherence [22, 24].

Consistent with the findings from several studies, the adolescents did not take their medication when they experienced side effects. Furthermore, the number, size, and taste of the drugs were barriers to adherence [18, 24, 25, 44]. Although the once daily fixed formulations facilitate adherence by reducing side effects [25], the adolescents in this study have already started the second-line treatment regimen because of treatment failure and would not benefit from fixed dose formulations.

As reported in other studies, adolescents were adhering because they had comprehensive understanding of the importance of taking ART and knowledge of the outcome of poor adherence. The adolescents desired to be healthy and normal and not be recognized as an HIV-positive individual. This motivated them to take their medication correctly to maintain their health and live longer [23, 26, 27, 45–47]. Being normal according to Mutwa et al. [23] means the absence of physical signs of living with HIV and also not being stigmatized. Since most ALPHIV initiate ART after prolonged illness, they attribute the improvements in their health and physical appearance to the ART medication [27, 45].

Onward self-disclosure to friends, teachers, and extended family members creates an additional supportive environment for adherence. Adolescents who disclosed their HIV status to friends and teachers had less difficulty in adhering to treatment and were able to make ART part of their daily life. Bernays et al. [48] argue that although disclosing HIV to children and adolescents is a critical step to improve or sustain adherence, it should not be interpreted as sufficient to ensure adequate adherence. In the current study, all the adolescents had disclosed their HIV status and yet 16 were not adhering. For disclosure to be truly beneficial to ALPHIV, there should be onward self-disclosure to friends, sexual partners, teachers, and other extended family members [45].

## 5. Limitations

The study used adolescent self-report to assess adherence which may not be accurate and could be influenced by social desirability; nevertheless, some of the adolescents were attending the failure clinic because of nonadherence. Secondly, we cannot conclude that the findings are representative of all ALPHIV in Francistown since ALPHIV were recruited from the IDCC only; however, because the IDCC is a referral centre, the adolescents were referred from various HIV centres across Francistown. Lastly, the study did not include the caregivers and guardians who are crucial in providing support or adherence and could have provided valuable information on adherence and the role of the family in ensuring adherence.

## 6. Conclusions

Fear of stigma and discrimination was one of the major barriers to adherence. Perceived stigma affected the time to take medication, the visit to the clinic for ART refill, and onward self-disclosure of HIV status to friends and partners. The fear of unintended disclosure and stigma were more pronounced in boarding schools and adolescents resorted to hiding and taking their medication in privacy. The heightened fear of being seen collecting ART medication affected keeping appointments for clinic visits. Stigma also influenced their choice of action when there was a clash between school activities and scheduled clinic appointments for ART refill.

The home as a supportive environment was the main facilitators for adherence and support was the strongest motivator for adolescents to adhere to and keep up with clinic appointments. Furthermore, self-disclosure created a supportive environment for adherence, particularly at school. Those who disclosed their HIV status experienced less challenges to adhere to ART medication. The desire to be healthy and live long was a major motivator for adherence at a personal level. This was influenced by understanding the benefits of ART and the consequences of poor adherence.

The fear of stigma shaped the adolescents' adherence to ART; therefore, healthcare providers need to address the fears adolescents have regarding stigma in boarding school and the school environment in general. An important strategy to reduce the fear of stigma is to encourage adolescents to self-disclose their HIV status to their close friends and partners since the fear of unintended disclosure fuelled perceived stigma. The involvement of the family in both stigma mitigation and onward self-disclosure cannot be overemphasised given the importance of the family and home environments in supporting and hindering ALPHIV adherence to ART. Furthermore, healthcare providers need to involve the adolescents in the planning of scheduled clinic appointments, consistent with realistic daily activities of adolescents.

## Figures and Tables

**Table 1:** Sociodemographic characteristics of adolescents with perinatal HIV.

Variables		Frequency	Percentage
*Age category*	12–15	15	50
	16–19	15	50

*Gender*	Male	17	57
	Female	13	43

*School grades*	Not attending school	3	10
	Primary level	9	30
	Secondary level	18	60

*Source of income*	Support from biological parents	19	63
	Support from guardian	11	37

*Living arrangements*	Living with single parent	9	30
	Living with both parents	10	33
	Living with guardian	11	37

**Table 2:** Emergent themes and subthemes.

Theme	Subtheme	Categories
*Facilitators*	Living arrangements	Support
		Clinic companion
		Treatment reminders
	Understanding the benefits of ART	
	Fear of getting sick and dying	
	Making ART a part of daily life	

*Barriers*	Lack of transport money	Household level
	Treatment side effects	Clinic level
	Clash between school and treatment schedule	School level
	Fear of stigma and discrimination	Personal level

## Data Availability

The qualitative data used to support the findings of this study are available from the corresponding author upon request.
